# Uneven host cell growth causes lysogenic virus induction in the Baltic Sea

**DOI:** 10.1371/journal.pone.0220716

**Published:** 2019-08-06

**Authors:** Nicole Köstner, Klaus Jürgens, Matthias Labrenz, Gerhard J. Herndl, Christian Winter

**Affiliations:** 1 Department of Limnology and Bio-Oceanography, Center of Functional Ecology, University of Vienna, Vienna, Austria; 2 Department of Biological Oceanography, Leibniz Institute for Baltic Sea Research (IOW), Rostock-Warnemünde, Germany; 3 NIOZ, Department of Marine Microbiology and Biogeochemistry, Royal Netherlands Institute for Sea Research, Utrecht University, AB Den Burg, The Netherlands; Stazione Zoologica Anton Dohrn, ITALY

## Abstract

In the Baltic Sea redoxcline, lysogenic viruses infecting prokaryotes have rarely been detected using the commonly used inducing agent mitomycin C. However, it is well known that not all viruses are induceable by mitomycin C and growing evidence suggests that changes in trophic conditions may trigger the induction of lysogenic viruses. We hypothesized that using antibiotics to simulate a strong change in trophic conditions for antibiotica-resistant cells due to reduced competition for resources might lead to the induction of lysogenic viruses into the lytic cycle within these cells. This hypothesis was tested by incubating prokaryotes obtained throughout the Baltic Sea redoxcline in seawater with substantially reduced numbers of viruses. We used a mixture of the protein synthesis-inhibiting antibiotics streptomycin and erythromycin to induce the desired changes in trophic conditions for resistant cells and at the same time ensuring that no progeny viruses were formed in sensitive cells. No inducible lysogenic viruses could be detected in incubations amended with mitomycin C. Yet, the presence of streptomycin and erythromycin increased virus-induced mortality of prokaryotes by 56–930% compared to controls, resulting in the induction of lysogenic viruses equivalent to 2–14% of *in situ* prokaryotic abundance. The results indicate the existence of a previously unrecognized induction mechanism for lysogenic viruses in the Baltic Sea redoxcline, as the mode of action distinctly differs between the used antibiotics (no virus production within affected cells) and mitomycin C (lysogenic viruses are produced within affected cells). Obtaining accurate experimental data on levels of lysogeny in prokaryotic host cells remains challenging, as relying on mitomycin C alone may severely underestimate lysogeny.

## Introduction

The Baltic Sea is the second largest brackish water system in the world, where a stable halocline between freshwater at the surface and saltier, deeper water is maintained through freshwater input from several large rivers [1]. A stable halocline restricts the import of oxygen (O_2_) from the surface into deeper water masses and also acts as a barrier for nutrient exchange. Consequently, deeper areas of the Baltic Sea are severely depleted in O_2_ due to respiration and contain high concentrations of hydrogen sulfide (H_2_S; [2,3]). The transition zone between the oxic surface layer and deeper anoxic waters is characterized by a stable redoxcline with steep gradients in O_2_, H_2_S, and high concentrations of nitrite, nitrate, phosphate, and ammonium [4,5]. Also, prokaryotic activity (the term prokaryotes here is used to denote members of the phylogenetic domains *Bacteria* and *Archaea*, no phylogenetic relationship is implied) is particularly high in the redoxcline, where chemolithoautotrophs perform dark CO_2_ fixation [6–8].

Protistan grazing and viral lysis constitute the two principal prokaryotic mortality factors in aquatic habitats [9,10]. In pelagic redoxclines, protistan grazing drastically declines below the oxic-anoxic interface, particularly when it becomes sulfidic [11,12]. However, also enhanced viral lysis could not be confirmed [11,13]. Previously, the Baltic Sea redoxcline was found to be an environment where high viral abundance in the order of 10^7^ viruses mL^-1^ is maintained by a combination of low prokaryotic virus production and low viral decay, resulting in exceptionally long viral turnover times of up to 84 d [13].

Viruses entirely depend on the metabolism of the host for proliferation and most viruses infecting prokaryotes, known as phages, fall into one of two groups: lytic and lysogenic viruses. Lytic viral infection directly leads to the production of viruses upon infection, followed by lysis of the host cell and release of progeny viruses into the environment. Lysogenic viruses may either directly enter the lytic cycle or at first form a symbiotic relationship with their host cell by integrating their genome into their host’s genome (prophage state) and remaining dormant before the lytic cycle is induced. Some lysogenic viruses can readily be induced into the lytic cycle by exposing the host cell to ultraviolet radiation or the chemical mitomycin C [14,15]. The cytostatic drug mitomycin C is mainly used in human cancer treatment. However, mitomycin C's effect on eukaryotes and prokaryotes is similar to the exposure to ultraviolet radiation: cells suffer from DNA-damage, preventing genome replication and cell division [16–18]. In prokaryotes, damage to the genomic DNA leads to the activation of the gene RecA, encoding for a DNA recombination and repair protein, as part of the SOS response, a cellular DNA repair mechanism that also may lead to the cleavage of certain phage repressor proteins responsible for maintaining some viruses in its lysogenic state [19].

Mitomycin C is widely used experimentally for inducing lysogenic viruses into the lytic cycle. Previously, mitomycin C was also used to screen the water column of the Baltic Sea for the presence of lysogenic viruses. An early study found that up to 80% of prokaryotic cells within the pelagic redoxcline were lysogenically-infected [20]. However, using a more refined incubation method, later studies concluded that lysogeny, if detectable at all by mitomycin C, is only of minor importance in the Baltic Sea water column [11,13]. This is in contrast to recent data obtained by the same method from the Arabian Sea showing that lysogeny can be as high as 48% in the suboxic zone and varying between 9–24% in the redoxcline [21]. Although DNA damage and the ensuing RecA-dependent SOS response are well known to induce many but not all lysogenic viruses into the lytic cycle [22,23], other RecA-independent induction mechanism have been described [24–27]. Also, lysogeny on a community level may be influenced by environmental parameters such as temperature or trophic conditions [28–32]. Thus, lysogenic viruses entering the lytic cycle upon induction due to RecA-independent mechanisms or due to changes in environmental conditions might still be present in the Baltic Sea redoxcline and possibly be missed by the mitomycin C-based approach.

Antibiotics constitute an effective way of altering the composition of a prokaryotic community. By inhibiting growth of susceptible members of the community, other not affected taxa might experience less competition for nutrients and, thus, a boost in growth. In that sense, the application of antibiotics to a mixed prokaryotic community alters the trophic conditions for a specific subset of the community by mitigating competition. For some environments, changes in the trophic conditions have been shown to induce the lytic cycle of lysogenic viruses [28,29,32]. Streptomycin and erythromycin are two antibiotics that interfere with protein synthesis. Streptomycin inhibits prokaryotic growth by binding to the 16S rRNA component of the 30S ribosomal subunit, altering the ribosome structure, resulting in tRNA mismatches and protein mistranslation [33]. Exposure to high concentrations of streptomycin may even lead to membrane permeabilization due to the insertion of mistranslated proteins into the cytoplasmic membrane [34]. Erythromycin binds to the 50S ribosomal subunit, mechanically blocking the ribosomal export tunnel and preventing peptide elongation [35]. Thus, viruses cannot be produced within streptomycin- and/or erythromycin-susceptible cells, because protein synthesis is inhibited also preventing the formation of new virus capsids. This is in contrast to mitomycin C-affected cells, which may be the source of progeny viruses due to induction of lysogenic viruses into the lytic cycle within these cells. Contrary to earlier studies [36–38], streptomycin as well as erythromycin have been shown to be effective against *Bacteria* as well as *Archaea* [39,40].

In this study we tested whether streptomycin and erythromycin can be used to experimentally induce lysogenic viruses infecting prokaryotes into the lytic cycle. We hypothesize that the growth-inhibiting effect of the antibiotics on susceptible taxa would result in boosted growth of the unaffected members of the prokaryotic community [41] due to reduced competition for nutrients. In turn, enhanced growth of some host cells might lead to the induction of lysogenic viruses into the lytic cycle within these cells without sustaining DNA damage. Thus, induction of lysogens in antibiotica-resistant host cells due to elevated growth would indicate the presence of lysogenic viruses that are inducible via a, as yet uncharacterized, RecA-independent induction mechanism.

## Materials and methods

### Study sites, sampling, and physicochemical parameters

Samples were taken at the oxic-anoxic interface at two stations in the Central Baltic Sea during a cruise with RV *Meteor* in June 2012. Three depth layers were sampled once at Gotland Deep (N 57°19.20' E 20°03.00', bottom depth: 248m) and twice at Landsort Deep (58° 34,998' N 18° 13,998' E, bottom depth: 460m). Based on the concentration of O_2_ and H_2_S, the samples covered the oxic zone (O_2_ > 30 μM, no H_2_S), the suboxic zone (30 μM ≥ O_2_ > 0 μM, no H_2_S), the transition zone (30 μM ≥ O_2_ > 0 μM, H_2_S > 0 μM), and the anoxic zone (no O_2_, H_2_S > 0 μM). For more details about the sampling stations and data on *in situ* prokaryotic and viral abundance as well as physicochemical parameters throughout the redoxcline see Köstner et al.[13].

### Experimental setup

For each sample, 1.8 L of water was filtered over 3 μm pore-size membrane filters (Cat. No. TSTP04700, 47 mm diameter; Merck Millipore, Darmstadt, Germany) to remove larger organisms and particles. Subsequently, two consecutive tangential flow filtration steps (Vivaflow 200, PES membrane, 0.2 μm pore size, Cat. No. VF20P7; Vivaflow 200, PES membrane, molecular weight cut-off 100 kDa, Cat. No. VF20P4, Sartorius Stedim Biotech, Göttingen, Germany) were performed to obtain a prokaryotic concentrate (size fraction of 0.2–3 μm; final volume ~100 mL) and ultra-filtered seawater (size fraction <100kDa) to be used as growth medium in the experiments. In total, nine experiments were performed, each with a control and three treatments in duplicate. The experiments are based on the knowledge that viruses are incapable of active movements, finding their host cells via a stochastic and density-dependent mechanism. By diluting viruses within a sample new virus infections are effectively prevented because the abundance of viruses is too low (virus dilution approach; [42]). Rising numbers of viruses over time within such incubations can only be the result of virus infections that have already occurred before sampling and setup of the experiments. This approach has already been applied to studying levels of virus-mediated mortality of prokaryotes throughout the Baltic Sea redoxcline [11,13]. Control treatments contained 5 mL of prokaryotic concentrate diluted in 45 mL of ultra-filtered seawater from the same sample. In addition, mitomycin C treatments (MI) contained 1 μg mL^-1^ of the drug, antibiotic treatments (STER) were amended with streptomycin (100 μg mL^-1^) and erythromycin (10 μg mL^-1^), and the fourth treatment contained mitomycin (1 μg mL^-1^), streptomycin (100 μg mL^-1^), and erythromycin (10 μg mL^-1^, MISTER). Incubations were performed in 60 mL glass vials equipped with air-tight butyl rubber seals and incubated in the dark at 4°C for 40 h. Sub-sampling for the enumeration of prokaryotes and viruses (see below) was performed at 5 h-intervals. To prevent oxygen contamination all sample handling, filtrations, and sub-sampling was performed in an anaerobic chamber filled with nitrogen gas.

### Enumeration of prokaryotes and viruses

Samples (1.8 mL) for determining prokaryotic abundance and viral abundance were fixed with glutaraldehyde (0.5% final concentration) for 10 min at room temperature before flash-freezing in liquid nitrogen and stored at -80°C. Upon thawing, prokaryotes and viruses were stained with SYBR Green I (final dilution: 1:20000 of 10000× commercial stock, Invitrogen, Life Technologies, Carlsbad, CA, USA) and enumerated on a BD FACSAria II flow cytometer (Becton Dickinson, Durham, NC, USA) as previously described [43,44].

### Determination of prokaryotic growth (PG), virus production (VP), and the frequency of infected cells (FIC)

Temporal changes in prokaryotic and viral abundance during the incubations were used to determine PG, VP, and FIC as previously described in detail by Köstner et al. [13]. In short, PG and VP were calculated from the positive slopes between local minima and maxima of prokaryotic and viral abundance, respectively (S1 Fig and S1 Table in [13]). In order to enable direct comparisons among treatments, all rate measurements (PG, VP) were corrected for differences between *in situ* and initial prokaryotic abundance at the start of the incubations. Likewise, FIC was calculated based on local minima and maxima of viral abundance and the prokaryotic abundance at the start of the experiments (S1 Fig and S1 Table in [13]). A constant burst size of 28 viruses per lysed host cell was assumed in FIC calculations [20]. Differences among treatments were assumed to be relevant when ranges of the duplicate incubations did not overlap. Thus, based on our data lysogeny is defined as the difference in FIC between a specific treatment and its corresponding control provided that FIC from the control is substantially lower compared to the treatment.

## Results

### Growth of prokaryotes

**Fig 1:**
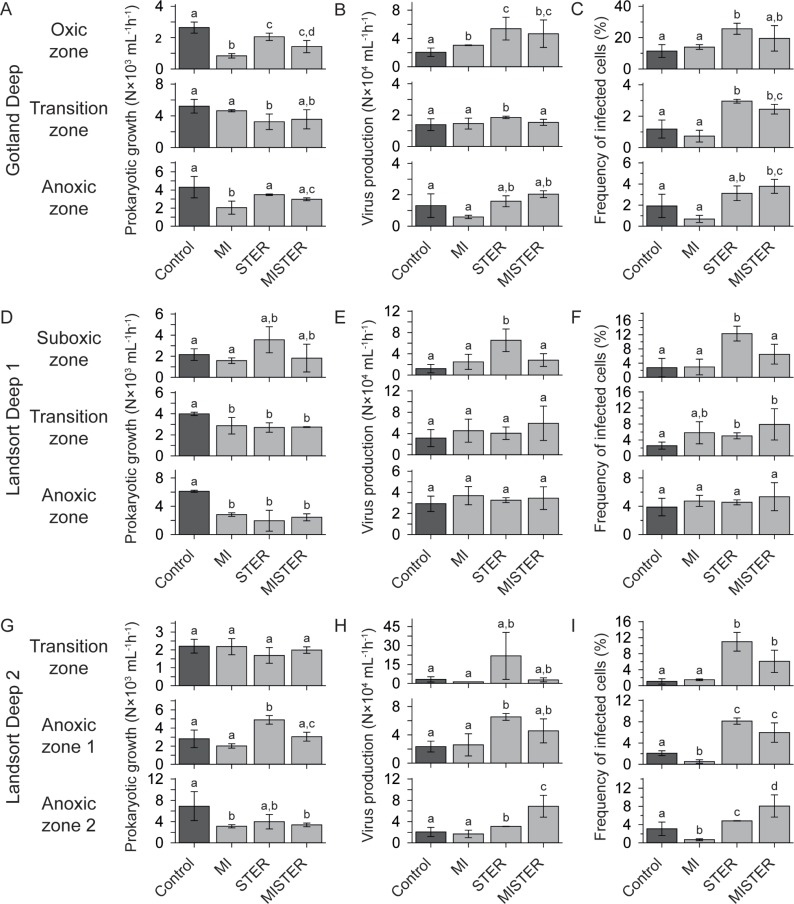
Treatment effects on prokaryotic growth (PG), viral production (VP) and the frequency of infected cells (FIC). The figure shows average values of duplicate incubations for PG (A, D, G), VP (B, E, H), and FIC (C, F, I) in controls and experimental treatments (MI: mitomycin C; STER: streptomycin and erythromycin; MISTER: mitomycin C, streptomycin, erythromycin) for each sampling station (Gotland Deep, Landsort Deep 1, Landsort Deep 2) and depth zone. Error bars show the range of duplicate incubations and lower-case letters indicate differences between treatments. Note the differences in *y*-axes scales.

Throughout all experiments and depth zones, average PG ranged from 2.2–6.9×10^3^ mL^-1^ h^-1^ in controls and from 0.8–4.9×10^3^ mL^-1^ h^-1^ in experimental treatments (MI, STER, MISTER; Fig 1A, 1D and 1G). Overall, PG in the treatments was either similar or 22–68% lower as compared to controls, with the exception of STER in the anoxic zone 1 at Landsort Deep 2 where it was 74% higher than in the corresponding control (Fig 1G). However, throughout all experiments and depth zones, PG was never negative in any of the treatments. PG in STER was either similar or 70–145% higher than in the MI treatments except for the transition zone at Gotland Deep where PG in STER was 30% lower than in MI (Fig 1A). In seven experiments PG did not differ between STER and MISTER. However, in the anoxic zone at Gotland Deep and the anoxic zone 1 at Landsort Deep 2, PG in MISTER was 15% and 38% lower than in STER, respectively, (Fig 1A and 1G).

### Viral proliferation

#### VP

Average VP in controls varied between 1.2–3.2×10^4^ mL^-1^ h^-1^ and between 0.6–21.8×10^4^ mL^-1^ h^-1^ in the treatments (Fig 1B, 1E and 1H). In the oxic zone at Gotland Deep VP in MI, STER, and MISTER was 48–127% higher compared to the control, yet in the anoxic zone none of the treatments had any effect on VP (Fig 1B). Also, VP in the transition zone at Gotland Deep was 33% higher in STER as compared to the control. At Landsort Deep 1, the only discernible treatment effect on VP was found in the STER treatment in the suboxic zone, where it was 440% higher compared to the control (Fig 1E). At Landsort Deep 2 VP in the anoxic zone 1 was 178% higher in STER than the control and in the anoxic zone 2 VP in STER and MISTER was 50% and 234% higher than the control, respectively (Fig 1H). All other treatments at Landsort Deep 2 were similar to the controls. Except for the transition and anoxic zone at Landsort Deep 1 where VP did not differ between MI and STER (Fig 1E), VP in STER was higher by 27–1596% compared to MI. In the transition zone at Gotland Deep and the suboxic zone at Landsort Deep 1, VP in MISTER was lower by 17% and 57% compared to STER, respectively (Fig 1B and 1E). In the anoxic zone 2 at Landsort Deep 2 VP in MISTER was 122% higher than in STER (Fig 1H). VP did not correlate with PG when calculated from all data or when only treatment data for each depth zone separately were taken into account (Spearman rank correlation, data not shown, in each case *p* > 0.05).

#### FIC

Throughout all control incubations, average FIC ranged from 1.1–11.4% of *in situ* prokaryotic abundance and in experimental treatments from 0.5–25.6% (Fig 1C, 1F and 1I). During seven experiments, the MI treatment had no effect on FIC as compared to the controls. However, in the anoxic zones 1 and 2 at Landsort Deep 2, FIC in MI was smaller than the controls by 75% and 78%, respectively (Fig 1I). In the anoxic zone at Gotland Deep (Fig 1C) and in the anoxic zone at Landsort Deep 1 (Fig 1F) FIC in STER did not change compared to controls. In all other experiments FIC in STER increased by 56–930% compared to controls. If data in a control incubation are substantially lower than data in the corresponding treatment (i.e., ranges of duplicates are not overlapping), levels of lysogeny can be calculated as the difference between treatment and control. Thus, our data translate into 1.7–14.2% of prokaryotic cells infected by lysogenic viruses. Also, FIC in STER was higher by 83–1441% compared to MI, except for the transition and anoxic zones at Landsort Deep 1 where it was similar to MI (Fig 1F). In MISTER, FIC was either similar or higher by 96–471% than the controls but never smaller. Also, in seven experiments FIC in MISTER and STER was similar. In the suboxic zone at Landsort Deep 1 FIC in MISTER was 47% smaller compared to STER (Fig 1F) and in the anoxic zone 2 at Landsort Deep 2 FIC in MISTER was 67% higher than in STER (Fig 1I). Similar to VP, FIC did not correlate with PG when calculated from all data or when only treatment data for each depth zone separately were taken into account (Spearman rank correlation, data not shown, in each case *p* > 0.05).

## Discussion

### Treatment effects on growth of prokaryotes

PG throughout all sampling stations and depth zones always was positive in every treatment, even in MISTER, where the presence of mitomycin C, streptomycin, and erythromycin challenged susceptible cells with inhibition of DNA replication and protein synthesis concomitantly (Fig 1A, 1D and 1G). Regardless of the differences in the mechanisms by which the used drugs act on prokaryotes [16,33,35], our data indicate that not all prokaryotic cells were affected equally by these treatments. In some cells DNA and/or protein synthesis might have been irreparably harmed, leading to cell death accompanied by the release of cell compounds into the ambient water [33]. Partially resistant cells might have been able to repair DNA-damage via the SOS-response, and/or degrade the antibiotics, and recover after some time of growth inhibition. A substantial fraction of prokaryotes in the Baltic Sea has been found to be resistant to antimicrobial agents [45,46] and, although growth of resistant cells is not directly affected by the presence of such drugs, it might be indirectly favored as resistant cells might take advantage of the suppression of potential competitors [41].

The lack of any correlation between PG and viral proliferation measured as VP and FIC (Fig 1 and results section) appears to contradict our initial hypothesis that reduction in competition due to susceptible cells suffering from antibiotic treatment should have increased growth of resistant cells that in turn were responsible for elevated viral proliferation in STER. However, given that our data represent bulk measurements of prokaryotic abundance and not single-cell production rates, enhanced growth of specific cells may be masked. At a minimum PG in the STER treatments indicates continued growth by a specific subset of the community together with no or reduced growth by other taxa. It has been demonstrated that uneven growth in prokaryotic communities may increase viral proliferation rates [47]. Indeed, in many cases VP was elevated together with increased FIC in the STER treatments (Fig 1).

Variation in PG among treatments was substantial, hence, no general trend in terms of treatment severity of the antimicrobial drugs could be identified (Fig 1). The lack of such a trend indicates substantial variability among sampled prokaryotic communities in their ability to withstand mitomycin C (MI), the combination of streptomycin and erythromycin (STER), or a cocktail of all three drugs (MISTER). One possible cause for this variability is that sulfate-reducing *Bacteria* found in the oxic-anoxic interface [48,49] may use produced H_2_S as defense mechanism against antibiotics [50]. This may be supported by our data as the variation in PG among treatments in all transition zone samples is exceptionally low compared to other depth zones (Fig 1A, 1D and 1G).

### Antimicrobial agents and their influence on viral proliferation

#### Mitomycin C

Lysogeny has long been thought to be the common viral replication strategy in environments with low host abundance and low activity while more productive systems with higher host abundances appear to favor lytic viruses [20,23]. This conclusion is mainly based on studies using mitomycin C as the inducing agent, nevertheless, not all lysogenic viruses can be induced into the lytic cycle by this substance [22,23]. A literature screening of relevant studies revealed that induction of lysogenic viruses into the lytic cycle by mitomycin C resulted in highly variable estimates for the fraction of lysogenic cells among environments with no consistent link to host density [51]. A finding that to a certain extent is also supported by our data from the MI treatments when comparing variability of PG and FIC (Fig 1A, 1D, 1G, 1C, 1F and 1I). Indeed, as FIC in MI treatments never was higher compared to the controls, induction of lysogenic viruses by mitomycin C was not detectable throughout the Baltic Sea redoxcline (see also [11,13]), while in many cases a clear treatment effect on PG was identifiable (Fig 1A, 1D and 1G).

#### Streptomycin and erythromycin

Streptomycin and erythromycin are inhibiting prokaryotic protein synthesis and, thus, also maturation of progeny viruses. Nevertheless, our data show that FIC in the STER treatments of seven experiments substantially increased by 56–930% compared to the controls (Fig 1C, 1F and 1I), often accompanied by increased VP (Fig 1B, 1E and 1H). Given that new infections during the time course of such incubations are prevented by dilution [13], these additional viruses (in comparison to the control) could only be due to viral infections that were already ongoing at the time of sampling. One might argue that these data might be caused by death and lysis of cells susceptible to streptomycin and erythromycin. Especially at high concentrations of streptomycin, susceptible cells may lyse due to the insertion of mistranslated proteins into the cell membrane and in case of current lytic viral infections this may lead to the release of progeny viruses before the end of the latent period [34,52]. Nevertheless, this mechanism does not seem plausible, because previously a concentration of 300 μg mL^-1^ of streptomycin was used to lyse cells within a one hour period (e.g., [52]), whereas in this study the 3×times lower concentration of 100 μg mL^-1^ was used. Even so, for the sake of argument let’s assume that all cells were susceptible to streptomycin-induced lysis at the beginning of the incubations (this is clearly not the case; Fig 1A, 1D and 1G) and that all virally-infected cells harbored mature progeny viruses at the end of the latent period. Based on FIC varying between 1.1–11.4% in the controls (Fig 1C, 1F and 1I), the frequency of virally infected cells containing mature progeny viruses can be calculated as 0.2–1.7% of *in situ* prokaryotic abundance [53]. However, elevated FIC in STER compared to controls translates into an additional fraction of 1.7–14.2% of *in situ* prokaryotic abundance that released viruses upon lysis. Thus, even with extreme and unrealistic assumptions, streptomycin-induced cell lysis cannot explain our data.

Another possible explanation for our findings is that burst size may have changed dramatically in STER treatments. In this study, FIC was calculated assuming a constant burst size of 28 viruses released for every lysed prokaryotic cell [20]. Given that the control and the treatments for each depth zone are derived from the same water sample, assuming a constant burst size is justified. Burst size is considered to be a virus taxon-specific trait, i.e., different viruses differ in their burst size [54]. Thus, increased FIC in STER treatments compared to controls may in principal be explained by a reduction in burst size. Yet given that new virus infections are prevented by the dilution of viruses during the incubations [13], this would indicate that a different set of virus taxa was lysing additional cells and that these viruses have already infected their host cells at the time of sampling. Regardless of whether or not a substantial change in burst size was the cause for elevated FIC in STER treatments or simply more viruses lysed more prokaryotic cells: both lines of interpretation require the presence of a different set of virus taxons compared to the control treatments, that were already present within their host cells at the time of sampling. The most plausible explanation for our data is that lysogenic viruses were induced into the lytic cycle. Given that the incubations were held in the dark and mitomycin C was not added to STER, the induction mechanism for these viruses likely was RecA-independent.

## Conclusions

Exposing prokaryotic communities from the Baltic Sea redoxcline to a mixture of the antibiotics streptomycin and erythromycin causes induction of lysogenic viruses into the lytic cycle. This method revealed that between 1.7–14.2% of prokaryotes contained lysogenic viruses in this environment, whereas mitomycin C-inducible prophages could not be detected (see also [11,13]). Contrary to the frequently-used inducing agent mitomycin C, streptomycin and erythromycin hinder protein synthesis within susceptible cells and do not cause DNA-damage. This implies that the induction mechanism does not depend on the cellular SOS-response. Instead, our data indicate that uneven growth of host cell populations (e.g., antibiotic-sensitive versus resistant cells) causes the observed induction of lysogenic viruses into the lytic cycle. Finally, relying solely on mitomycin C may severely underestimate the fraction of prokaryotic cells infected by lysogenic viruses.
